# PPAR-gamma Fun(gi) With Prostaglandin

**DOI:** 10.1177/1550762919899641

**Published:** 2020-01-23

**Authors:** Robert J. Evans, Simon A. Johnston

**Affiliations:** 1 The Francis Crick Institute, London, UK; 2 The University of Sheffield, Sheffield, UK

**Keywords:** *Cryptococcus* neoformans, fungal infection, host pathogen interactions, macrophages, zebrafish, eicosanoids, 15-keto-PGE_2_, PPAR-gamma

## Abstract

In our recent publication, we show for the first time that the fungal pathogen *Cryptococcus neoformans* is able to manipulate host cells by producing eicosanoids that mimic those found in the host. Using complementary in vivo zebrafish and in vitro macrophage cell culture models of *Cryptococcus* infection, we found that these eicosanoids manipulate host innate immune cells by activating the host receptor PPAR-gamma which is an important regulator of macrophage inflammatory phenotypes. We initially identified PGE2 as the eicosanoid species responsible for this effect; however, we later found that a derivative of PGE2—15-keto-PGE_2_—was ultimately responsible and that this eicosanoid acted as a partial agonist to PPAR-gamma. In this commentary, we will discuss some of the concepts and conclusions in our original publication and expand on their implications and future directions.

Comment on: Evans RJ, Pline K, Loynes CA, Needs S, Aldrovandi M, Tiefenbach J, Bielska E, Rubino RE, Nicol CJ, May RC, Krause HM, O’Donnell VB, Renshaw SA, Johnston SA. 15-keto-prostaglandin E2 activates host peroxisome proliferator-activated receptor gamma (PPAR-γ) to promote Cryptococcus neoformans growth during infection. PLoS Pathog. 2019 Mar;15(3):e1007597. doi: 10.1371/journal.ppat.1007597. eCollection 2019 Mar. PubMed PMID: 30921435; PubMed Central PMCID: PMC6438442.

*Cryptococcus neoformans* is a pathogenic fungus that is ubiquitous in our everyday environment, but only those with severe or unusual immune deficiencies, such as HIV AIDS, develop serious disease.^1^ During infection *Cryptococcus* forms a close interaction with host macrophages—after phagocytosis by macrophages *Cryptococcus* is able survive and replicate within the phagosome, subverting macrophage function and turning the macrophage into a niche for the establishment of infection.^2^ To kill *Cryptococcus*, macrophages must be activated by a Th1 CD4+ T-cell-mediated adaptive immune response (hence the prevalence in HIV AIDS patients)^3^; failure to control intracellular infection can lead to dissemination from the lungs into the central nervous system and the development of fatal fungal meningitis.

*Cryptococcus* can influence the activation state of infected macrophages, shifting them from protective Th1 activation states to a nonprotective Th2 state although the biological mechanisms behind this are unclear.^4^ Our hypothesis for this study was that this manipulation might be mediated by eicosanoid species produced by the fungus.^5^ *Cryptococcus* can produce a number of eicosanoid species which closely resemble those found in the host but natural purpose of these lipids normally associated with cell to cell signaling in multicellular organisms is unknown.^6^ Macrophages and other innate immune cells are highly responsive to eicosanoid species such as prostaglandins and leukotrienes so we reasoned that eicosanoids produced by the fungus during intracellular infection could interfere with normal host signaling pathways.

## Quantifying Eicosanoids During *Cryptococcus* Infection and Determining Their Source

Very little is known about eicosanoid synthesis pathways in *Cryptococcus*; only two *Cryptococcus* enzymes—phospholipase B1 (*PLB1*) and laccase (*LAC1*)—have been linked to eicosanoid synthesis in the fungus.^7,8^ The lack of homologs to eicosanoid synthesis enzymes found in higher organisms suggests that the pathway is distinct from anything previously described. Deletion mutants for *PLB1*^8^ and *LAC1*^9^ have been characterized in *Cryptococcus*. The *PLB1* mutant (*Δplb1*) shows a profound decrease in all eicosanoids produced by *C neoformans* suggesting this enzyme is central to eicosanoid synthesis—possibly fulfilling a role analogous to phospholipase A_2_ in mammalian cells. The *LAC1* mutant (*Δlac1*) is deficient in only PGE_2_ and its derivative 15-keto-PGE_2_, suggesting this enzyme might fulfill a role analogous to prostaglandin E_2_ synthase in mammalian cells. Both of these strains were used in our study to differentiate between host- and pathogen-derived eicosanoids; to aid the study of these strains in our zebrafish model, we produced green fluorescent protein–tagged versions of each strain. *Δplb1* is known to have a growth defect in macrophages,^10^ whereas laccase activity has been found to positively correlate with increased mortality in patients with HIV-associated cryptococcosis^11^—although how much this is due to PGE_2_ synthesis as opposed to the role of laccase in the production of another cryptococcal virulence factor melanin. In our study, we were able to rescue the in vitro intracellular proliferation defect of *Δplb1* with exogenous PGE_2_; we also found that both *Δplb1* and *Δlac1* had reduced in vivo growth in our zebrafish larvae cryptococcosis model; however, only *Δplb1*-infected fish responded to exogenous PGE_2_ or 15-keto-PGE_2_.^5^ We attribute *Δ*lac1’s unresponsiveness to exogenous prostaglandin treatment to the fact that laccase is also responsible for aforementioned melanin synthesis—thus, it is possible that for this strain both melanin and PGE_2_ are required for wild-type levels of growth—or an unknown defect that was responsible for it being much more attenuated in animal infection than the *Δplb* mutant. We attempted to circumvent this difference by disrupting the macrophage immune response but found that any immunocompromise of this response was critical to survival and confounded any differences.^12^

A major challenge we faced in our study was measuring eicosanoid levels during host-pathogen interactions and determining whether the eicosanoids measured were host or pathogen derived. A previous study by Shen and Liu^13^ found that pulmonary levels of PGE2 increased in mice infected with *C neoformans*; however, they were unable to attribute this to host or pathogen production. In our study, we performed experiments to measure differences in PGE_2_ content between wild-type H99 and *Δplb1*-infected macrophages using 2 different methods—ELISA (enzyme-linked immunosorbent assay) and LC MS/MS (liquid chromatography-tandem mass spectrometry). We found that J774 macrophages did produce detectable levels of PGE_2_; however, we did not see any significant difference between infected or uninfected macrophages or between H99, *Δplb1* and *Δplb1:PLB1*-infected cells (although the concentrations detected with ELISA and LC MS/MS were very similar).^5^ This suggested that *Cryptococcus*-derived eicosanoids present during infection were likely to be contained within the macrophage. Measurement of these small, localized eicosanoid levels within infected macrophages proved very difficult with current analytical methods—to our knowledge, intracellular levels of pathogen-derived eicosanoids have never been quantitatively measured before. To overcome this difficulty, we took a different approach; we used a co-infection assay which has previously been used to investigate the interaction of different *Cryptococcus gattii* strains within the same macrophage.^14^ We predicted that the parental cryptococcal strain produced growth-promoting eicosanoids but *Δplb1* could not; the *Δplb1* strain should display improved intracellular replication when H99 is also present within the same macrophage. Indeed, we found that *Δplb1* proliferated better when accompanied by 2 wild-type yeast cells in the same macrophage as opposed to when 2 *Δplb1* yeast cells were accompanied by 1 wild-type yeast cell. These experiments confirmed to us that *Cryptococcus* produced eicosanoids during macrophage infection and suggested that they did remain contained within the macrophage—important because it indicated that any host receptor targeted was likely to be intracellular.

## Identifying a Mechanism

**Figure 1.**
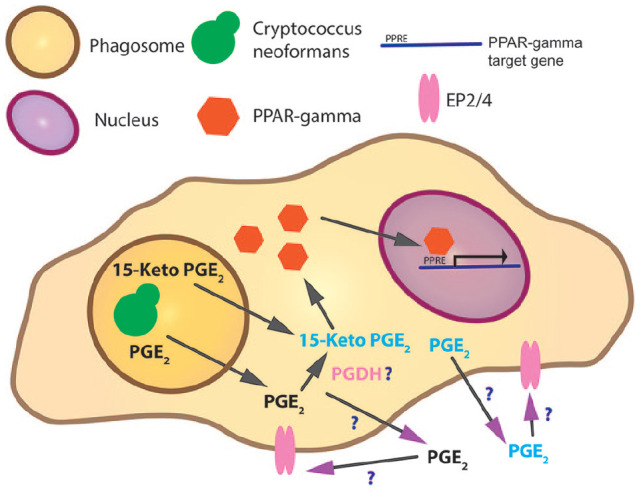
Evidenced and potential pathways of eicosanoid and PPAR-gamma interactions during *Cryptococcus* infection of macrophages. *Note.* During infection, *Cryptococcus* resides within the phagosome. Prostaglandin E_2_ or 15-keto-PGE_2_ is produced by the fungus. Generation of 15-keto-PGE_2_ is either by *Cryptococcus* or the host—or perhaps both. 15-Keto PGE_2_ is a partial agonist to PPAR-gamma. PPAR-gamma is a cytosolic eicosanoid receptor; on ligand binding and activation, PPAR-gamma translocates to the nucleus where it binds to and activates genes with a PPRE target motif. In addition, prostaglandin E_2_ may bind its extracellular receptors EP2/4 on macrophages or other cells. PPAR = peroxisome proliferator–activated receptor; PPRE = peroxisome proliferator hormone response elements; PGDH = 15-hydroxy prostaglandin dehydrogenase.

Our initial experiments indicated that PGE_2_ was the eicosanoid species required for *Cryptococcus* growth because exogenous addition of this species was sufficient to rescue the growth defects of *Δplb1* in J774 macrophages and zebrafish larvae. Intending to boost the observed effects of PGE_2_, we used a chemically altered version of PGE_2_ called 16,16 dimethyl PGE_2_ that cannot be metabolized.^15^ To our surprise, the opposite outcome occurred—16,16-dimethyl PGE_2_ could no longer rescue the growth of *Δplb1*. Under physiological conditions, PGE_2_ can be further converted to 15-keto-PGE_2_ by the enzyme 15-hydroxy prostaglandin dehydrogenase (PGDH; Figure 1).^16^ We assumed that conversion from PGE_2_ to 15-keto-PGE_2_ could be a way for the host to mitigate the effects of *Cryptococcus-*derived (or exogenously added) PGE_2_. This was a eureka moment for our study because we realized that conversion of PGE_2_ into 15-keto-PGE_2_ was actually required for the growth of *Cryptococcus* and that if host eicosanoid signaling was being affected it was through a 15-keto-PGE_2_ receptor rather than a PGE_2_ receptor.

Our experiments had shown that *Cryptococcus*-derived 15-keto-PGE_2_ promoted cryptococcal growth and that any host receptors involved were likely to be intracellular (Figure 1). While searching for putative receptors, we found that 15-keto-PGE_2_ had been reported to be an agonist for peroxisome proliferator–activated receptor gamma (PPARγ)—an intracellular receptor that is known to control inflammatory responses. PPARγ is a cytosolic receptor that has a variety of ligands including many lipid eicosanoids. Ligand binding leads to the formation of a heterodimer between PPARγ and retinoid X receptor (RXR). Following heterodimer formation, the PPARγ/RXR complex translocates to the nucleus and acts as a transcription factor controlling the expression of genes which possess a peroxisome proliferator hormone response element.^17^

Through in vivo experiments with a transgenic zebrafish PPARγ reporter, we found that 15-keto-PGE_2_ was unable to activate the PPARγ reporter itself; however, when 15-keto-PGE_2_ was added in combination with a full PPARγ agonist troglitazone, the level of PPARγ activation was reduced compared with a troglitazone-only control. This indicated that 15-keto-PGE_2_ could interact with PPARγ in some capacity either as a partial agonist (a partial agonist is an agonist that binds to a receptor with a weak affinity and as a result does not fully activate the receptor) or an antagonist. To resolve this question, we proved that the effects of 15-keto-PGE_2_ were reversed by a known PPARγ antagonist. From these data, we concluded that 15-keto-PGE_2_ is a partial agonist to PPARγ, a finding that is supported by a previous study^18^ (Figure 1). Interestingly, we settled on this conclusion through interpretation of our data and it was only afterward that we became aware of other partial agonists against PPARγ.^19,20^ The protein structure of PPARγ has evolved to provide different binding sites for full and partial agonists within the PPARγ ligand-binding domain (LBD)—full agonists bind to and stabilize the H12 alpha-helix of the LBD which produces a binding site for PPARγ transactivators. In contrast, partial agonists do not interact with the H12 alpha-helix and as a result do not provide stabilization of this region but binding still produces PPARγ activation to varying magnitudes.^21^ Partial agonism is a mechanism that allows great flexibility in transcription factor function, rather than modulating the full gamut of PPARγ-controlled genes, a partial agonist will only activate a subset of these genes. This means a receptor like PPARγ can produce a variety of different transcriptional responses depending on the partial agonists present.

## Future Perspectives

1. Where is PGE_2_ metabolized into 15-keto-PGE_2_ during infection? PGE_2_ is quickly metabolized into 15-keto-PGE_2_ in living cells (Figure 1). In higher organisms, this reaction is performed by PGDH. It is therefore possible that PGE_2_ produced by *Cryptococcus* is metabolized into 15-keto-PGE_2_ by the host. 15-keto-PGE_2_ has been detected in the supernatant of *Cryptococcus* cultures so it is also likely that *Cryptococcus* possesses an enzyme similar in function to PGDH.
2. What is the effect of PPARγ activation by 15-keto-PGE_2_ on host cells—specifically host macrophages? We have found that 15-keto-PGE_2_ is a partial agonist to PPARγ; this means that agonist binding only modulates a subset of PPARγ-controlled genes (Figure 1). Identifying this subset in host cells will be essential to understand how 15-keto-PGE_2_ enables *Cryptococcus* to cause infection.
3. Do other *Cryptococcus*-derived eicosanoids promote virulence? Our study has focused on PGE_2_/15-keto-PGE_2_ production by *Cryptococcus*. We also tested PGD_2_ but found this had no effect on infection. *Cryptococcus* produces many more eicosanoids which could have synergistic effects to PGE_2_/15-keto-PGE_2_ or completely different effects. In view of our findings, future studies in this area should also consider metabolites which could be produced from *Cryptococcus* eicosanoids within the host.
